# Enhancing carotenoid biosynthesis in rice endosperm by metabolic engineering

**DOI:** 10.1111/pbi.13059

**Published:** 2019-02-18

**Authors:** Yong‐Sheng Tian, Bo Wang, Ri‐He Peng, Jing Xu, Tong Li, Xiao‐Yan Fu, Ai‐Sheng Xiong, Jian‐Jie Gao, Quan‐Hong Yao

**Affiliations:** ^1^ Shanghai Key Laboratory of Agricultural Genetics and Breeding Biotechnology Research Institute of Shanghai Academy of Agricultural Sciences Shanghai China; ^2^ State Key Laboratory of Crop Genetics and Germplasm Enhancement College of Horticulture Nanjing Agricultural University Nanjing China

**Keywords:** rice, carotenoids, β‐carotene, multigene transformation, co‐expression

Most cereal grains contain small amounts of dietary carotenoids. Thus, vitamin A deficiency (VAD) is common in third‐world countries where the nutrition (of the population) depends on a single staple crop such as rice. Thus, considerable research effort has been dedicated to achieving sustainable and economical production of carotenoids in crops (Paine *et al*., 2005). Plant carotenoids are isoprenoid‐derived molecules, and isoprenoids are produced via the mevalonate (MVA) or plastidial 2‐*C*‐methyl‐D‐erythritol 4‐phosphate (MEP) routes, both giving rise to isopentenyl diphosphate (IPP) (Hemmerlin *et al*., 2012). However, little is known about the influence of the genes involved in the MVA or MEP pathways on carotenoid production in transgenic plants.

3‐Hydroxy‐3‐methylglutaryl coenzyme A reductase (HMGR) is generally considered the major rate‐limiting enzyme in the MVA pathway. Transgenic tomatoes over‐expressing plant HMGR without also over‐expressing the genes encoding phytoene synthase (*Psy*) and phytoene desaturase (*CrtI*) show IPP accumulation and no carotenoid (over‐) accumulation (Enfissi *et al*., 2005). To further investigate the effect of HMGR (overexpression) on carotenoid pathways in rice endosperm, we heterologously expressed *Psy* from maize (*ZmPsy*) and *CrtI* from *Pantoea ananatis* (*PaCrtI*) along with *tHMG1*, which encodes truncated HMGR from *Saccharomyces cerevisiae*, in this study. The three genes (*tHMG1*,* ZmPsy1* and *PaCrtI1*) were chemically synthesized through PCR‐based two‐step DNA synthesis (PTDS) (Xiong *et al*., 2006). The codons of these genes were optimized and preferentially designed for rice. Then, a gene expression cassette (G*tHMG1*, G*ZmPsy1* and G*PaCrtI1*) was constructed by inserting the ORF between an endosperm‐specific rice globulin‐1 promoter and terminator using the PAGE‐mediated overlap extension PCR method (Peng *et al*., 2014). In this way, different restriction sites were added to the 5′ and 3′ ends of the expression cassette. The starting point for the construction of the plasmid for rice transformation was pCAMBIA‐1301, and the vector was modified through the introduction of new multiple cloning sites to yield the plant binary vector pYP694 (Peng *et al*., 2014). Then, pYP69‐G*tHMG1* was prepared by inserting the G*tHMG1* expression cassette into pYP694 at the *Eco* RI and *Bam* HI sites, and G*ZmPsy1* was inserted into the pYP69‐G*tHMG1* vector at the *Bam* HI and *Kpn* I sites to obtain the two‐gene construct pYP69‐G*tHMG1*‐G*ZmPsy1*. Finally, the three‐gene construct (pYP69‐G*tHMG1*‐G*ZmPsy1‐GPaCrtI1*, genotype HPC) was obtained using the *Kpn* I and *Xba* I sites of the abovementioned two‐gene construct and named pYR2420. (Fig.** 1a). The two‐gene construct (pYP69‐G*ZmPsy1‐GPaCrtI1*, genotype PC) was obtained using the same method mentioned above and named pYR2421.

**Figure 1:**
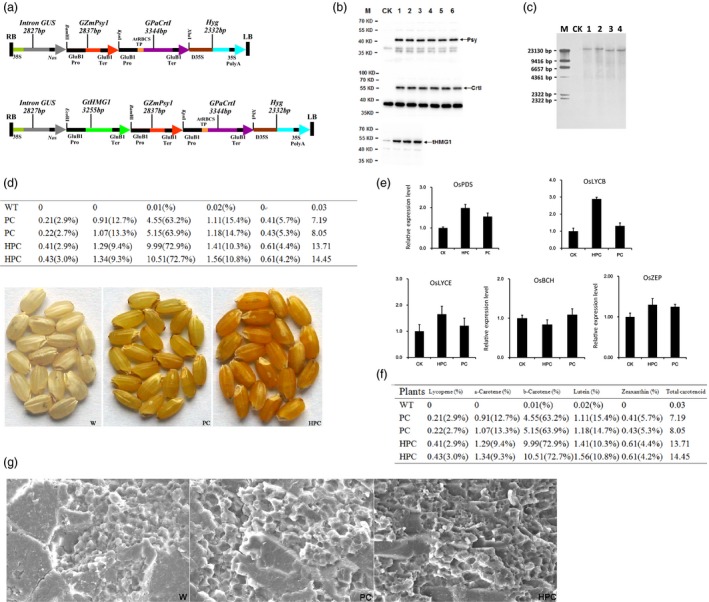
Enhancing carotenoid biosynthesis in rice endosperm. (a) Schematic representation of the recombinant vectors used for rice transformation. Upper: PC construction containing two genes; Lower: HPC construction containing three genes. (b) Western blot analyses of PC and HPC transgenic seed proteins. W, wild‐type; 1, 2 and 3, different lines with the HPC construction containing three genes; 4, 5 and 6, different lines with the PC construction containing two genes. (c) Genomic Southern blot analyses of HPC (1, 2) and PC (3, 4) transgenic rice plants.(d) Seed carotenoid content and composition of PC and HPC transgenic rice plants (μg/g dry weight) and comparison of representative seed colour phenotypes among nontransgenic wild‐type (W) rice and independent PC and HPC transgenic rice lines at the T_4_ generation. (e) Expression of endogenous carotenogenic genes in transgenic rice endosperm. Relative transcript levels of endogenous genes in T_4_ rice endosperms at 25 DAP expressing different transgenic combinations. Values are the means ± SD of three quantitative real‐time PCR replicates. (f) The agronomic traits of PC (1,2,3) and HPC (4,5,6) transgenic rice plants. (g) Scanning electron microscope images of transverse sections of mature rice seeds. W: wild‐type; PC: expressing *ZmPSY1* and *PaCRTI*; HPC: expressing *tHMG1*,* ZmPSY1* and *PaCRTI*. Scale bars: 50 μm.

Thus, we transformed seven‐day‐old mature zygotic rice embryos of japonica‐type (*Oryza sativa* sub sp. *japonica* ‘wuyun No. 8’) with the recombinant vectors pYR2420 and pYR2421 by *A*grobacterium‐mediated direct DNA transfer. The generated T_1_ seeds with the most intense yellow endosperms were used for the generation of T_2_ plants. This process was repeated for the T_4_ generation. Subsequently, the results of genomic DNA polymerase chain reaction (PCR) and RT‐PCR demonstrated that the inserted genes were actively and stably transcribed in the transgenic plants. To further assess whether the proteins produced by the three transgenes were correctly expressed, we performed Western blot analysis of the seeds using antibodies specific for the transgenic proteins. A cleaved Psy protein of 46.5 kDa and a CrtI protein of 55 kDa were clearly detected in the HPC and PC lines, but no protein was detected in wild‐type controls. In the HPC transgenic lines, a tHMG1 protein of 48.4 kDa was detected (Fig.** 1b). Our results indicated that the transgenes were not silenced and that the resulting individual protein components had been correctly expressed. The combined results clearly demonstrated that the three genes had stable and coordinated expression in transgenic rice.

The transgenic HPC lines produced more yellow endosperms than the PC transgenic lines, indicating increased carotenoid accumulation. Previous studies have demonstrated that transgenic rice plants with a more intense yellow endosperm resulted from high transgene copy numbers (Bai *et al*., 2015). Therefore, genomic Southern blots were performed using specific probes to determine transgene copy numbers in HPC and PC rice. Two HPC and two PC transgenic lines were analysed using the same probes, revealing the presence of a single copy of the transgene in all plant lines (Fig. 1c). The main objective of this study was to engineer rice grains to improve carotenoid abundance in the rice endosperm. We then measured the total content and composition of carotenoids in the same T_4_ seeds through HPLC analysis. We found that HPC lines accumulated an average of 14.2 μg/g dry weight of total carotenoids in their endosperms. This value was approximately one‐fold higher than the average total carotenoid level in the PC lines (Fig.** 1d). Although the content of total carotenoids and each carotenoid in different studies varies with respect to the experimental conditions, comparing the percentage of each carotenoid as a proportion of the total content is useful. HPC endosperms contained higher levels of individual carotenoids, including β‐carotene (1.1‐fold), zeaxanthin (67%), α‐carotene (27%) and lutein (27%), than the PC endosperms. Our results demonstrated that the HPC construct was more efficient than the PC construct in driving the production of β‐carotene and total carotenoids (Fig.** 1d).

The enhanced accumulation of carotenoids in transgenic plants was associated with the up‐regulation of endogenous carotenoid biosynthetic genes (Huang *et al*., 2015). Thus, we examined the expression of endogenous carotenogenic genes, including phytoene desaturase (OsPDS), lycopene ε‐cyclase (OsLYCE), β‐carotene hydroxylase (OsBCH2) and zeaxanthin epoxidase (OsZEP), by RT‐qPCR upon their putative regulation as a result of transgene expression. In transgenic HPC plants, the transcripts of *OsPDS*,* OsLYCE*,* OsLYCB* and *OsZEP* were increased between 0.2‐ and 1.9‐fold relative to their expression in PC and WT plants (Fig.** 1e). The highest levels (increased by 1.9‐fold) were found for the *OsLYCB* gene in the endosperms of the HPC rice plants, correlating with an increased β‐carotene content. In contrast, the transcript levels of *OsBCH* were lower in HPC plants than in PC and WT plants, correlating with the high accumulation of β‐carotene instead of lutein and zeaxanthin.

Unpredictable negative effects on host plants have been reported in many attempts at metabolic engineering in plants (Farré *et al*., 2014). Thus, the agronomic traits of transgenic rice under field conditions were investigated. The phenotypic evaluation of transgenic and non‐transgenic wild‐type (WT) plants revealed no major difference at the vegetative growth stage. The yield parameters were also scored to investigate whether the expression of the three genes in endosperms affected the grain yield of transgenic rice plants under field conditions (Ha *et al*., 2010). Figure** 1f shows that the grain yield of the transgenic lines remained similar to that of the WT controls under normal field conditions. Overall, these results indicated that carotenoid production in the endosperm does not affect the normal growth and grain yield of the transgenic plants.

Starch accounts for approximately 80% of the total dry matter content of the rice endosperm, and the composition and content of starch as well as the starch granule structure in the endosperm have an important influence on rice quality. Therefore, the starch structure was analysed by scanning electron microscopy. The endosperm of transgenic rice contained some closely arranged irregularly spherical or ellipsoidal starch granules, as observed in the endosperm of WT plants (Fig.** 1g). These results demonstrated that the starch granules in the transgenic rice endosperm were well formed and arranged evenly at the centre of the rice grain.

In conclusion, the expression of *tHMG1* combined with *ZmPSY1* and *PaCRTI* significantly enhanced the accumulation of carotenoids in rice endosperm by boosting the flux through the MVA pathway and creating a metabolic sink towards carotenoids. Our methods, combining three primary genes responsible for β‐carotene biosynthesis into one vector and introducing it into rice plants by *A*grobacterium‐mediated direct DNA transfer (Zhu *et al*., 2009), are simple and less expensive than conventional methods, such as successive rounds of crosses between transgenic lines or sequential retransformation. Overall, our transgenic rice can be used as a functional grain that can contribute to the mitigation of VAD and as a raw material for the production of β‐carotene from ‘green plant factories’.

## Conflict of interest

The authors declare no conflicts of interest.
